# Single-Cell Differential Network Analysis with Sparse Bayesian Factor Models

**DOI:** 10.3389/fgene.2021.810816

**Published:** 2022-02-04

**Authors:** Michael Sekula, Jeremy Gaskins, Susmita Datta

**Affiliations:** ^1^ Department of Bioinformatics and Biostatistics, University of Louisville, Louisville, KY, United States; ^2^ Department of Biostatistics, University of Florida, Gainesville, FL, United States

**Keywords:** Bayesian, factor model, scRNA-seq, gene co-expression network, differential network analysis

## Abstract

Differential network analysis plays an important role in learning how gene interactions change under different biological conditions, and the high resolution of single-cell RNA (scRNA-seq) sequencing provides new opportunities to explore these changing gene-gene interactions. Here, we present a sparse hierarchical Bayesian factor model to identify differences across network structures from different biological conditions in scRNA-seq data. Our methodology utilizes latent factors to impact gene expression values for each cell to help account for zero-inflation, increased cell-to-cell variability, and overdispersion that are unique characteristics of scRNA-seq data. Condition-dependent parameters determine which latent factors are activated in a gene, which allows for not only the calculation of gene-gene co-expression within each group but also the calculation of the co-expression differences between groups. We highlight our methodology’s performance in detecting differential gene-gene associations across groups by analyzing simulated datasets and a SARS-CoV-2 case study dataset.

## 1 Introduction

Gene network modeling has become essential to the understanding of complex biological systems related to health and disease. These networks allow researchers to uncover and interpret relationships and interactions between genes during different biological processes (Blencowe et al., 2019). There are several popular methods for constructing gene networks from microarray and bulk RNA sequencing data (Margolin et al., 2006; Langfelder and Horvath, 2008; Huynh-Thu et al., 2010), and more recently, methods for identifying gene networks from single-cell RNA sequencing (scRNA-seq) data have also been proposed (Specht and Li, 2016; Chan et al., 2017; Matsumoto et al., 2017; Sekula et al., 2020). Interestingly, the vast majority of these methods have focused only on analyzing gene expressions from one cellular population, such as a single tissue type, disease, or environmental condition.

Since biological systems are highly dynamic, there is also great interest in performing differential network analysis to examine the changes in network structure under different biological settings. In the context of bulk population data (i.e., microarray and bulk RNA sequencing), efforts have been made to develop different strategies for identifying differences between gene-gene networks. Some approaches propose qualitative analyses through visual inspection of different network topologies (Caldana et al., 2011; Weston et al., 2011), while others rely on statistical tests to determine differences across conditions (Choi and Kendziorski, 2009; Gill et al., 2010; Fukushima, 2013).

For scRNA-seq data, some research has been focused on providing guidelines and procedures for differential network analysis based on existing methods that have been developed to analyze different types of transcriptome data (e.g., bulk RNA sequencing, microarray). Cui et al. (2021) propose a pipeline for comparing two single-cell clusters that includes differential gene correlation analysis from McKenzie et al. (2016), weighted correlation network analysis from Langfelder and Horvath (2008), and differential network analysis with the method DiffCoEx (Tesson et al., 2010). Wang et al. (2017) present several proof-of-concept analyses of scRNA-seq data to identify genes that are differentially connected across distinct biological conditions by utilizing a differential connectivity test originally developed by Gill et al. (2010) for microarray gene expression data.

Some research has also been focused in developing new methods designed specifically for scRNA-seq data to identify and compare gene networks from two (or more) biological conditions. In Chiu et al. (2018), a differential network analysis method for scRNA-seq data is proposed that first determines a sample size corrected gene-gene correlation matrix for each cellular state and then identifies differential gene-gene pairs across the states. Ye et al. (2020) use co-expression network analysis and subgraph learning to identify interactive gene groups within subpopulations of cells from scRNA-seq data. Both Dai et al. (2019) and Li et al. (2021) propose novel methods to create cell-specific networks to examine the overall associations between genes for each individual cell. From these cell-specific networks, researchers can further identify changes in gene-gene networks across different cellular populations and/or different time points.

In this work, we propose a hierarchical Bayesian factor model for constructing gene co-expression networks (GCNs) from scRNA-seq data to explore differences in the network structure across various cell groups due to different biological conditions, cell types, cell stages, or other group choice. Treatment-dependent parameters in our model determine which latent factors are activated in a gene, thereby allowing for the calculation of gene-gene co-expression within each treatment group. For simplicity, we consider a two-group setting and refer to these groups as treatment and control, but our model can easily be extended to a multiple group scenario, if necessary.

The rest of this manuscript is organized as follows. We define our proposed model and inference for differential network analysis in Section 2. Results from simulation studies and real data analysis are presented in Section 3 to demonstrate the performance of our methodology. In Sections 4 and 5, we conclude with a discussion on our results and findings.

## 2 Methods

### 2.1 Hierarchical Bayesian Factor Model for Two Treatment Groups

Let *Y*_*gi*_ be the expression count of gene *g* (*g* = 1, …, *G*) in cell *i* (*i* = 1, …, *N*) for treatment *t*_*i*_ ∈ {0, 1}, where *t*_*i*_ = 0 represents that cell *i* belongs in the control (reference) group and *t*_*i*_ = 1 for the treatment group. We assume that each expression comes from the Poisson(*μ*_*gi*_) distribution (conditionally) and model the log-mean *log*(*μ*_*gi*_) through the representation

$$\log\left(\mu_{gi}\right)=\beta_{g}+t_{i}\delta_{g}+\overset{F}{\underset{f=1}{\sum }}\lambda_{if}\alpha_{gf;t_{i}}-\left\{\overset{F}{\underset{f=1}{\sum }}\frac{\alpha_{gf;t_{i}}^{2}}{2}\right\}.$$ (1)

For each cell *i*, there are *F* associated latent factors ***λ***_***i***_ = {*λ*_*i*1_, *…*, *λ*_*iF*_} that impact the expression. Each factor can be thought of as some unique cellular attribute (e.g., cell stage, pseudotime point) that will only affect a specific set of related gene expressions. Since we are defining our model on the log scale, we assume these factors come from a Normal(0, 1) distribution. Marginally over ***λ***_***i***_, the parameter *β*_*g*_ denotes the log-mean expression for gene *g* in the control group, and *β*_*g*_ + *δ*_*g*_ is the log-mean expression for gene *g* in the treatment group. Hence, *δ*_*g*_ represents the log-fold change in the expression for gene *g*.

The magnitude of the impact of factor *f* on gene *g* in treatment *t* is influenced by the parameter $\alpha_{gf;t}\in R$ . With this setup, the expression for gene *g* in treatment *t* is minimally impacted by factors with *α*_*gf*;*t*_ values close to 0 and greatly impacted by factors with absolute values of *α*_*gf*;*t*_ much greater than 0. It is important to note that the *α*_*gf*;*t*_’s are treatment-dependent which allows factors to impact the gene expressions differently across the treatments. Clearly, if *α*_*gf*;0_ and *α*_*gf*;1_ have similar values, then factor *f* has a similar influence on the gene expression in both treatments. However, the more interesting case is when *α*_*gf*;0_ and *α*_*gf*;1_ have very different values, which indicates a difference in the impact of factor *f* on gene *g* between the groups. By examining the differences between the $\alpha_{t}={\left\{\alpha_{gf;t}\right\}}_{\left(g,f\right)}$ matrices, we can identify differences between the gene networks of the treatment groups.

For most factors, we assume that the values of *α*_*gf*;0_ and *α*_*gf*;1_ in our model will be similar. That is, we expect *α*_*gf*;0_ and *α*_*gf*;1_ to be similar for most (*g*, *f*) pairs. We also expect each factor *f* to impact only a small number of genes, and so the ***α***_***t***_ matrices will be sparse. To that end, we define the following hierarchy on the *α*_*gf*;*t*_ parameters:

$$\begin{array}{cc} \alpha_{gf;t} & ∼Normal\left({\tilde{\alpha }}_{gf},\kappa_{gf;t}^{2}\tau_{f}^{2}\right),\\ \kappa_{gf;t} & ∼half-Cauchy\left(0,1\right),\\ \tau_{f} & ∼half-Cauchy\left(0,1\right), \end{array}$$ (2)

$$\begin{array}{cc} {\tilde{\alpha }}_{gf} & ∼Normal\left(0,\zeta^{2}\right),\\ \zeta & ∼half-Cauchy\left(0,1\right), \end{array}$$ (3)

where *half−Cauchy*(0, 1) is the standard half-Cauchy distribution with the probability density function

$$p\left(x\right)=\frac{2}{\pi \left(1+x^{2}\right)},\;x>0.$$

We refer to this model definition as Sparse Factor Model - Single HorseShoe (SFM-SHS). Under this scheme, the horseshoe prior (Carvalho et al., 2009) placed on each *α*_*gf*;*t*_ in Eq. 2 will help shrink the values of *α*_*gf*;0_ and *α*_*gf*;1_ together toward the common value ${\tilde{\alpha }}_{gf}$ . For a given factor *f*, we define *τ*_*f*_ as the global shrinkage parameter and the *κ*_*gf*;*t*_’s as the local shrinkage parameters. The global shrinkage parameter will pull the values of *α*_*gf*;0_ and *α*_*gf*;1_ toward ${\tilde{\alpha }}_{gf}$ across *g* = 1, *…*, *G*, while the treatment-dependent local shrinkage parameters will allow some values to be much different than ${\tilde{\alpha }}_{gf}$ . Thus, the *κ*_*gf*;*t*_’s can account for any variability between the groups. Our model favors borrowing information across treatments, so it should be efficient for factor-gene effects that are common. Nevertheless, the horseshoe priors allow big differences to accommodate differences between treatments.

To achieve more sparsity, a horseshoe prior could also be placed on the ${\tilde{\alpha }}_{gf}$ parameters to help shrink most of these values close to 0. To that end, we may replace Eq. 3 in our model with the following:

$$\begin{array}{cc} {\tilde{\alpha }}_{gf} & ∼Normal\left(0,\omega_{gf}^{2}\zeta^{2}\right),\\ \omega_{gf} & ∼half-Cauchy\left(0,1\right),\\ \zeta & ∼half-Cauchy\left(0,1\right). \end{array}$$ (4)

We refer to this second model definition as Sparse Factor Model - Double HorseShoe (SFM-DHS). Here, *ζ* is a global shrinkage parameter that will pull the values of ${\tilde{\alpha }}_{gf}$ toward 0. In Eq. 4, we introduce local shrinkage parameters (*ω*_*gf*_’s) to allow some of the ${\tilde{\alpha }}_{gf}$ values to be much different than 0. Therefore, the horseshoe priors on the *α*_*gf*;*t*_ parameters (Eq. 2) will promote sparsity in the treatment difference and the horseshoe priors on the ${\tilde{\alpha }}_{gf}$ parameters (Eq. 4) will promote sparsity in the underlying common network.

The flexibility of our defined factor structure allows for the zero-inflation and high cell-to-cell variability typical of scRNA-seq data. For a given factor *f*, the latent *λ*_*if*_ is unique to each cell *i* and only affects a particular gene within a treatment when *α*_*gf*;*t*_ ≠ 0. If the activated factors *λ*_*if*_ *α*_*gf*;*t*_ for a given gene are highly negative, then *μ*_*gi*_ will be very small and account for the high proportion of zeros typical of this data. Conversely, large positive values of the factors will increase *μ*_*gi*_ (relative to the baseline of either *exp*{*β*_*g*_} for the control group or *exp*{*β*_*g*_ + *δ*_*g*_} for the treatment group) and yield extremely large counts, i.e., overdispersion. In Eq. 1, the adjustment term of $-\left\{\sum_{f=1}^{F}\frac{\alpha_{gf;t_{i}}^{2}}{2}\right\}$ is included in our model to ensure that *E*[*Y*_*gi*_] in the control group is equal to *exp*{*β*_*g*_} and *E*[*Y*_*gi*_] is equal to *exp*{*β*_*g*_ + *δ*_*g*_} for the treatment group (after marginalizing out ***λ***_***i***_) regardless of the *α*_*gf*;*t*_ values. While we choose to let *Y*_*gi*_ follow a Poisson distribution conditional on the ***λ***_***i***_ terms, the variance of *Y*_*gi*_ (marginal on ***λ***_***i***_) is

$$\begin{array}{cc} Var\left[Y_{gi}\right]=exp\left\{\beta_{g}+t_{i}\delta_{g}\right\}\left[1+exp\left\{\beta_{g}+t_{i}\delta_{g}\right\}\overset{F}{\underset{f=1}{\prod }}\left(exp\left\{\alpha_{gf;t}^{2}\right\}-1\right)\right]\;,\\ Var\left[Y_{gi}\right] & >exp\left\{\beta_{g}+t_{i}\delta_{g}\right\}\left[1\right]=E\left[Y_{gi}\right]. \end{array}$$ (5)

Hence, *Y*_*gi*_ is conditionally Poisson but marginally overdispersed.

To complete the specification of our Bayesian model, we define priors for the average gene expression parameters as $\beta_{g}∼\;Normal\left(0,\sigma_{\beta }^{2}\right)$ and $\delta_{g}∼\;Normal\left(0,\sigma_{\delta }^{2}\right)$ , with standard deviation hyperparameters *σ*_*β*_ and *σ*_*δ*_ from half-Cauchy(0, 1). Our methodology does rely on the tuning parameter *F*, a fixed number of latent factors that is often unknown. Nevertheless, one can fit multiple models with different numbers of factors and choose the most suitable model based on comparing the estimated number of differential edges between the different choices of *F*. As discussed in more detail later in Section 3.1, we found that the overall results will remain relatively consistent for different choices of the tuning parameter *F*.

### 2.2 Network Structure

This model uses the parameters *α*_*gf*;*t*_ to characterize the relationship between the genes and a set of latent factors; however, our real interest is in using these parameters to learn about the genes themselves (marginally over these factors). While the ***α***_***t***_ matrices in our model impose a crude network structure on the gene expressions for each treatment, the individual *α*_*gf*;*t*_ parameters are non-identifiable, and so we cannot perform inference about these parameters directly. To that end, we consider the matrices $A_{t}=\alpha_{t}\alpha_{t}^{T}$ whose elements are identifiable.

For a given treatment *t*, the (*g*, *g*′) element (*g* ≠ *g*′) of the *G* × *G* matrix ***A***_***t***_ provides a summation of impact by the associated factors that are active in both genes *g* and *g*′ since $A_{t\left(g,g^{\prime }\right)}=\sum_{f=1}^{F}\alpha_{gf;t}\alpha_{g^{\prime }f;t}$ . This expression also happens to be equal to the covariance (after marginalizing out ***λ***_***i***_) between the values of  *log*(*μ*_*gi*_) and *log*(*μ*_*g*′*i*_) in treatment *t*,

$$\mathrm{Cov}\left[\log\left(\mu_{gi}\right),\log\left(\mu_{g^{\prime }i}\right)\right]=\overset{F}{\underset{f=1}{\sum }}\alpha_{gf;t}\alpha_{g^{\prime }f;t}.$$

With the marginal variance for *log*(*μ*_*gi*_) being

$$\mathrm{Var}\left[\log\left(\mu_{gi}\right)\right]=\overset{F}{\underset{f=1}{\sum }}\alpha_{gf;t}^{2},$$

the correlation between *log*(*μ*_*gi*_) and *log*(*μ*_*g*′*i*_) is defined as

$$Corr\left[\log\left(\mu_{gi}\right),\log\left(\mu_{g^{\prime }i}\right)\right]=\rho_{gg^{\prime };t}=\frac{\overset{F}{\underset{f=1}{\sum }}\alpha_{gf;t}\alpha_{g^{\prime }f;t}}{\sqrt{\left(\overset{F}{\underset{f=1}{\sum }}\alpha_{gf;t}^{2}\right)\left(\overset{F}{\underset{f=1}{\sum }}\alpha_{g^{\prime }f;t}^{2}\right)}}.$$ (6)

We focus our interest on the marginal correlation of the log-means due to the simplistic nature of the correlation structure and its reliance on only the *α*_*gf*;*t*_ parameters. As displayed in Eq. 5, the variance expression of *Y*_*gi*_ includes a set of *β*_*g*_ and *δ*_*g*_ parameters that cannot be factored out, which means the correlation structure between *Y*_*gi*_ and *Y*_*g*′*i*_ will depend on the average expression for each gene in each treatment. For this reason, we do not utilize the correlation structure between *Y*_*gi*_ and *Y*_*g*′*i*_.

### 2.3 Network Inference

Our methodology is coded in Stan (Stan Development Team, 2020), and the usual approach to Bayesian inference in Stan is to generate samples from the posterior distributions with Hamiltonian Monte Carlo (HMC; Neal, 2011). The estimated gene-gene network structure ${\tilde{N}}_{t}={\left\{{\tilde{n}}_{gg^{\prime };t}\right\}}_{\left(g,g^{\prime }\right)}$ within each treatment group is obtained by analyzing the posterior of the marginal correlation matrix in Eq. 6. To provide a quantifiable value of the association between genes *g* and *g*′ within treatment *t*, *M* samples of each (*g*, *g*′) element in the correlation matrix are used to calculate the posterior mean ${\hat{\rho }}_{gg^{\prime };t}=\frac{1}{M}\sum_{m=1}^{M}\rho_{gg^{\prime };t}^{\;\left(m\right)}\;$ . Additionally, the credible interval (CrI) of the posterior is examined to determine whether or not genes *g* and *g*′ are associated with one another within each treatment group, separately. For a given level of significance *α* ^∗^, two genes will have a significant association when zero is excluded from the 100(1 − *α* ^∗^)*%* CrI. To rank correlations by significance within each treatment group, the smallest a^∗^ such that the 100(1 − a^∗^)*%* CrI includes 0 for the given gene-gene pair can be determined. The corresponding a^∗^ value indicates the proportion of the posterior distribution outside of the smallest CrI that includes 0, which can be viewed as an approximate “*p*-value”.

When performing differential network analysis, the interest is in examining the difference between *ρ*_*gg*′;0_ and *ρ*_*gg*′;1_ (*θ*_*g* *g*′_ = *ρ*_*gg*′;0_ − *ρ*_*gg*′;1_), and both the posterior mean ${\hat{\theta }}_{gg^{\prime }}=\frac{1}{M}\sum_{m=1}^{M}\left(\rho_{gg^{\prime };0}^{\;\left(m\right)}-\rho_{gg^{\prime };1}^{\;\left(m\right)}\right)$ and the 100(1 − *α* ^∗^)*%* CrI for each gene-gene pair correlation difference are obtained from the posterior. If zero is excluded from the 100(1 − *α* ^∗^)*%* CrI, the difference between the treatment correlations for gene *g* and gene *g*′ is significant. An approximate “*p*-value” can also be determined and used to rank the differences in correlation between the treatment groups.

We note that an iterative Markov Chain Monte Carlo (MCMC) approach may be computationally intensive for larger scRNA-seq datasets and will require the user to perform various diagnostic checks to ensure MCMC convergence and mixing (Cowles and Carlin, 1996). For those reasons, we present an alternative strategy for inference of our model parameters. A posterior mode estimate from our model can be obtained by maximizing the joint posterior via the *optimizing* function from Stan. With this optimized estimate, we calculate the marginal correlation structure from each treatment group defined in Eq. 6 and determine each gene-gene pair correlation difference ${\hat{\theta }}_{gg^{\prime }}={\hat{\rho }}_{gg^{\prime };0}-{\hat{\rho }}_{gg^{\prime };1}$ . The ${\hat{\theta }}_{gg^{\prime }}$ values can be ranked based on their magnitudes to determine the gene-gene pairs whose correlations are most different at the posterior mode, but the significance of each difference cannot be directly determined from a single optimization.

To produce an estimate of variability for *θ*_*gg*′_, we utilize a nonparametric bootstrap procedure. For each bootstrap iteration (*m*_*b*_) we perform the following:

1) Resample the cellular data with replacement within each treatment group. Here, the number of cells within each treatment group remains the same, but a new sample of cellular data for each treatment group is randomly selected from the original treatment group data.
2) Obtain a posterior mode estimate from the resampled data with the *optimizing* function from Stan.
3) Determine the marginal correlation structure as defined in Eq. 6 for each treatment and then calculate ${\hat{\theta }}_{gg^{\prime }}^{\;m_{b}}={\hat{\rho }}_{gg^{\prime };0}^{\;m_{b}}-{\hat{\rho }}_{gg^{\prime };1}^{\;m_{b}}$ .

**FIGURE 1:**
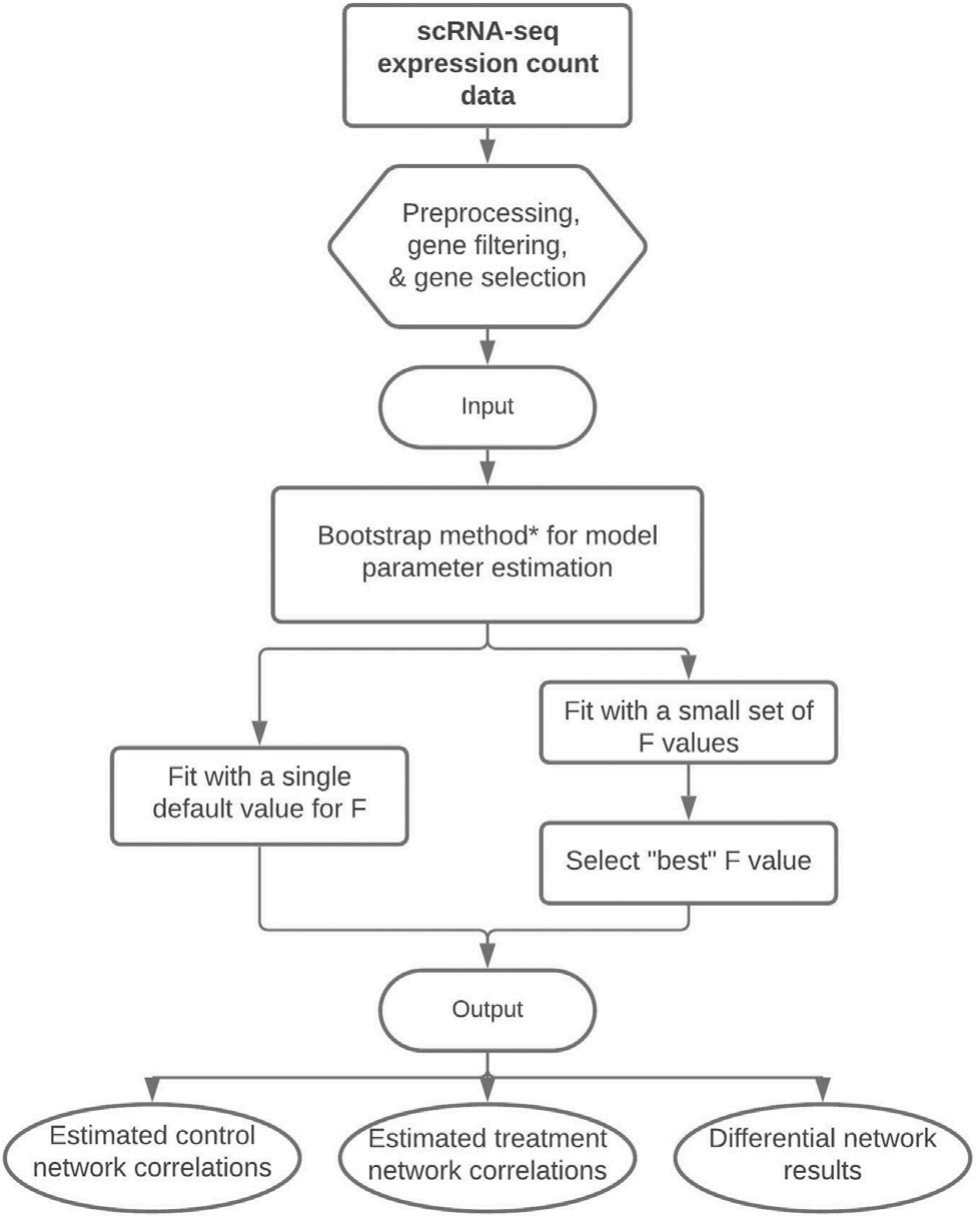
Flowchart of our proposed methodology to perform differential network analysis from scRNA-seq data. The input is a preprocessed scRNA-seq dataset and the output consists of the estimated control and treatment gene-gene correlations and the differential network with significant gene-gene differences between treatment groups. *Note: The bootstrap method is our recommended technique for model parameter estimation, but parameter estimation could alternatively be performed with a single posterior optimization or via HMC.

From the *M*_*b*_ total bootstrap samples, a 100(1 − *α* ^∗^)*%* confidence interval (CI) can be created and analyzed for *ρ*_*gg*′;0_, *ρ*_*gg*′;1_, and *θ*_*gg*′_ in the same manner as previously described above for the posterior CrIs. Figure 1 displays a flowchart of our proposed methodology. We note that we describe three estimation approaches/algorithms for our methodology, each with varying levels of computational complexity. The nonparametric bootstrap procedure is our preferred approach as will be shown in Section 3.

## 3 Results

### 3.1 Simulation Studies

To evaluate the performance of our methodology, we simulated count data from marginal zero-inflated negative binomial distributions via the NORmal To Anything (NORTA) algorithm (Cario and Nelson, 1997). The NORTA algorithm generates a random vector from a multivariate standard normal distribution with a given correlation structure and transforms it into a random vector with a specified marginal distribution. Counts were generated with the *rnorta* function from the R package SimCorMultRes (Touloumis, 2016), and the ZIM package (Yang et al., 2018) was used to estimate the parameters of the zero-inflated negative binomial distributions from genes randomly selected from the genes considered in a previous analysis (Sekula et al., 2020) of the mouse microglia cell data from Tay et al. (2018). It is important to emphasize that the simulated data come from a different generating model than our proposed estimation approach.

We considered four different simulation schemes to create datasets of different sizes, using either *G* = 50 or *G* = 100 genes and setting the total number of cells to either *N* = 500, *N* = 1, 000, or *N* = 2, 000 (see Table 1 for details of each simulation scheme). To define treatment groups, the cells were divided equally into the control group (*t* = 0) and the treatment group (*t* = 1). Correlation structures for each treatment network were fixed to create a differential structure of 325 different edges with *G* = 50 genes (Sim 1, Sim 2, and Sim 3) and 1,300 different edges with *G* = 100 genes (Sim 4).

**TABLE 1:** Comparison of the “true” differences between networks and the estimated differences between networks in the simulation studies for each differential network method. Results displayed for SFM-SHS and SFM-DHS are from the bootstrap estimation procedure. In Sim 1–3, there are 325 “true” differential edges and in Sim 4 there are 1, 300 “true” differential edges.

Sim 1: *G* = 50, *N* = 1, 000				
**Network Structure A**	**TPR**	**FDR**	**AUROC**	**Diff. Edges**
**SFM-SHS;** *F* = 8	0.782	0.000	0.980	254
**SFM-DHS;** *F* = 10	0.809	0.123	0.966	300
**DGCA**	0.825	0.056	0.935	284
**scdNet**	0.908	0.187	0.956	363
**Network Structure B**				
**SFM-SHS;** *F* = 7	0.855	0.007	0.992	280
**SFM-DHS;** *F* = 13	0.825	0.118	0.963	304
**DGCA**	0.858	0.021	0.983	285
**scdNet**	0.680	0.208	0.861	279
**Sim 2: ** ***G* = 50**, ***N* = 500**				
**Network Structure A**	**TPR**	**FDR**	**AUROC**	**Diff. Edges**
**SFM-SHS;** *F* = 7	0.732	0.040	0.942	248
**SFM-DHS;** *F* = 8	0.840	0.099	0.946	303
**DGCA**	0.892	0.020	0.955	296
**scdNet**	0.908	0.117	0.966	334
**Network Structure B**				
**SFM-SHS;** *F* = 7	0.874	0.004	0.979	285
**SFM-DHS;** *F* = 12	0.855	0.045	0.963	291
**DGCA**	0.849	0.028	0.942	284
**scdNet**	0.628	0.143	0.872	238
**Sim 3:** ***G* = 50**, ***N* = 2, 000**				
**Network Structure A**	**TPR**	**FDR**	**AUROC**	**Diff. Edges**
**SFM-SHS;** *F* = 8	0.871	0.000	0.997	283
**SFM-DHS;** *F* = 8	0.985	0.140	0.983	372
**DGCA**	0.945	0.130	0.978	353
**scdNet**	0.972	0.296	0.983	449
**Network Structure B**				
**SFM-SHS;** *F* = 7	0.898	0.000	0.999	292
**SFM-DHS;** *F* = 8	0.985	0.075	0.998	346
**DGCA**	0.957	0.116	0.986	352
**scdNet**	0.954	0.213	0.981	394
**Sim 4:** ***G* = 100**, ***N* = 1, 000**				
**Network Structure A**	**TPR**	**FDR**	**AUROC**	**Diff. Edges**
**SFM-SHS;** *F* = 8	0.922	0.000	0.997	1,199
**SFM-DHS;** *F* = 8	0.958	0.162	0.970	1,487
**DGCA**	0.875	0.023	0.972	1,165
**scdNet**	0.941	0.184	0.976	1,498
**Network Structure B**				
**SFM-SHS;** *F* = 7	0.964	0.025	0.994	1,285
**SFM-DHS;** *F* = 10	0.850	0.137	0.974	1,280
**DGCA**	0.883	0.042	0.970	1,199
**scdNet**	0.734	0.159	0.889	1,135

**FIGURE 2:**
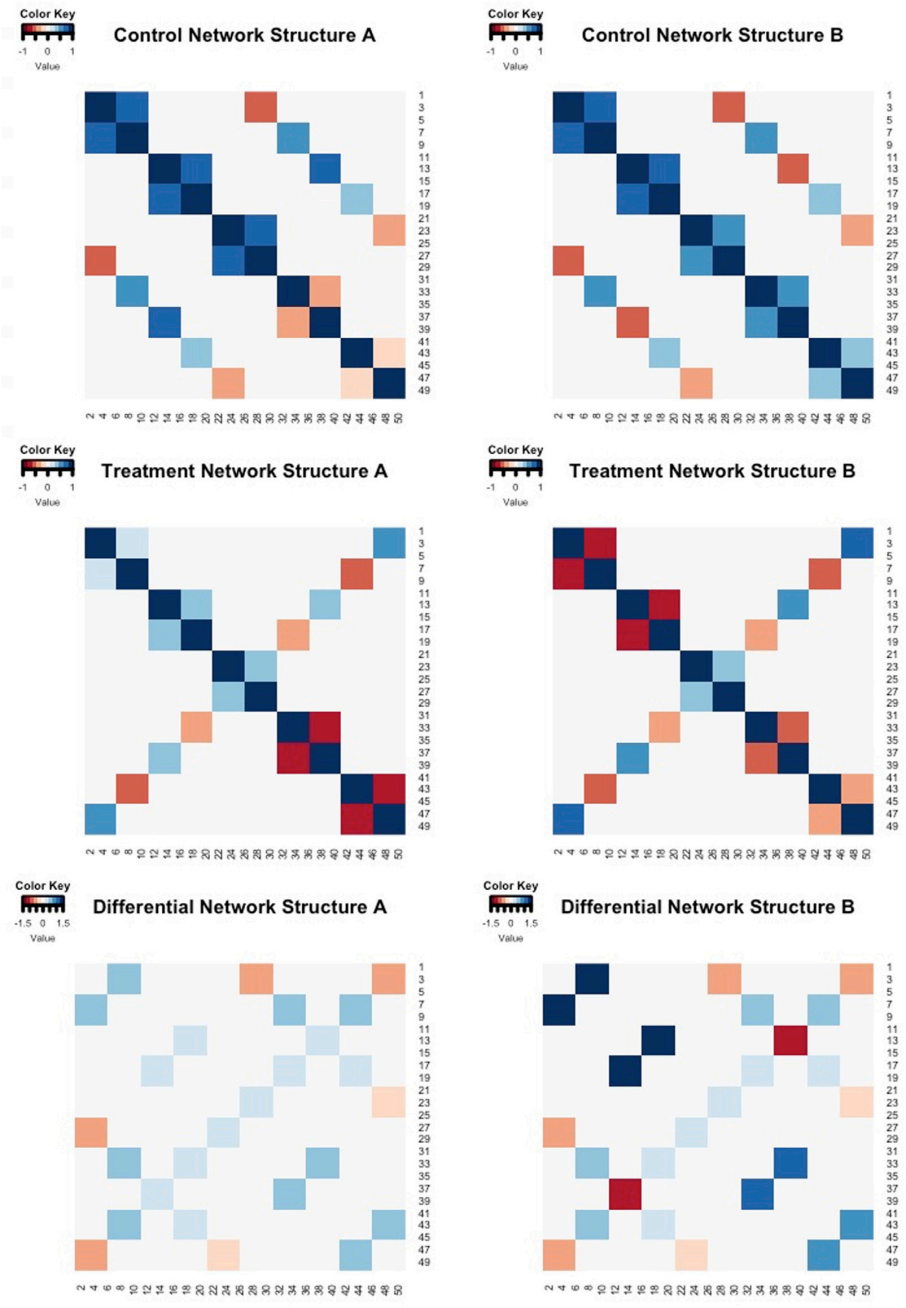
Heatmaps of the “true” correlation structures of control group (row 1) and treatment group (row 2) for Network Structure A and Network Structure B. The colored cells in these heatmaps represent the magnitude and direction of gene-gene correlations. Row 3 contains the differences between the control and treatment correlation structures. The colored cells in row 3 indicate the magnitude and direction of the differences in gene-gene associations across the two groups (control - treatment).

In our simulation data, we control the correlation structures by sorting genes into ten equal groups (e.g., Group 1 consisted of the first set of *G*/10 genes, Group 2 consisted of the second set of *G*/10 genes). Genes within the same group are defined to be highly correlated and share correlation structures across the other gene groups. Two different sets of correlation structures (Network Structure A and Network Structure B) were utilized for each simulation scheme, and the magnitudes of the “true” differences between correlations (*θ*_*gg*′_ = *ρ*_*gg*′;0_ − *ρ*_*gg*′;1_) ranged from 0.27 to 1.43 as displayed in Figure 2. For Network Structure B, some of the gene-gene pairs were simulated to have opposite correlation directions in each treatment group, thereby creating larger correlation differences between the groups.

Two versions of our sparse Bayesian factor methodology were investigated in the simulation studies. In our first model version SFM-SHS, the priors defined in Eqs. 2, 3 are placed on the *α*_*gf*;*t*_ and ${\tilde{\alpha }}_{gf}$ parameters, respectively (i.e., we use horseshoe priors on the *α*_*gf*;*t*_’s but not on the ${\tilde{\alpha }}_{gf}$ ’s). Recall that this encourages similarity between ***α***_***0***_ and ***α***_***1***_, but does not encourage sparsity in the shared base network $\tilde{\alpha }$ . For the second model version SFM-DHS, the prior on each ${\tilde{\alpha }}_{gf}$ parameter in Eq. 3 is replaced with the horseshoe prior defined in Eq. 4.

Using the simulated data, we ran our proposed models in R (R Core Team, 2018) interfacing with Stan through the package rstan (Stan Development Team, 2020). Here, we utilize the bootstrap procedure for parameter estimation and inference was performed on 1,000 bootstrap samples. To investigate whether the number of factors (*F*) makes any impact on model performance, we ran both models multiple times and input a different number of factors for each run, starting with *F* = 5 and increasing the number of factors up until *F* = 20. From our simulation studies, we found that area under the receiver operating characteristic curve (AUROC) for the performance of our models is relatively consistent with choices of *F* that are greater than 5, see Table 2. The inverse of the approximate “*p*-value” (defined in Section 2.3) for each ${\hat{\theta }}_{gg^{\prime }}$ was used for the AUROC calculations of SFM-SHS and SFM-DHS. When determining true positive rate (TPR) and false discovery rate (FDR) for our methods, we utilized the 95% CIs of the difference between each *ρ*_*gg*′;0_ and *ρ*_*gg*′;1_ pair for each dataset (*θ*_*gg*′_).

**TABLE 2:** Example comparisons of AUROC and number of significant differential edges (Diff. Edges) from Sim 1 when different choices of *F* are input into the SFM-SHS and SFM-DHS methods. Results are presented from the bootstrap estimation procedure.

	Sim 1: *G* = 50, *N* = 1, 000			
	Network structure A		Network structure B	
**SFS-SHS**	**AUROC**	**Diff. Edges**	**AUROC**	**Diff. Edges**
*F* = 5	0.886	217	0.888	278
*F* = 7	0.996	228	0.992	**280**
*F* = 8	0.980	**254**	0.984	260
*F* = 10	0.983	228	0.983	266
*F* = 12	0.981	222	0.979	256
*F* = 13	0.979	215	0.982	253
*F* = 15	0.980	224	0.973	253
*F* = 18	0.974	212	0.979	255
*F* = 20	0.973	197	0.981	232

	Network structure A		Network structure B	
**SFS-DHS**	**AUROC**	**Diff. Edges**	**AUROC**	**Diff. Edges**
*F* = 5	0.882	210	0.892	287
*F* = 7	0.990	242	0.982	292
*F* = 8	0.969	280	0.976	292
*F* = 10	0.966	**300**	0.974	283
*F* = 12	0.960	300	0.973	298
*F* = 13	0.946	288	0.963	**304**
*F* = 15	0.955	292	0.956	296
*F* = 18	0.952	272	0.960	280
*F* = 20	0.951	282	0.960	279

The number in bold font denotes the “peak” of the differential edges across the different number of factor choices for each network structure.

**FIGURE 3:**
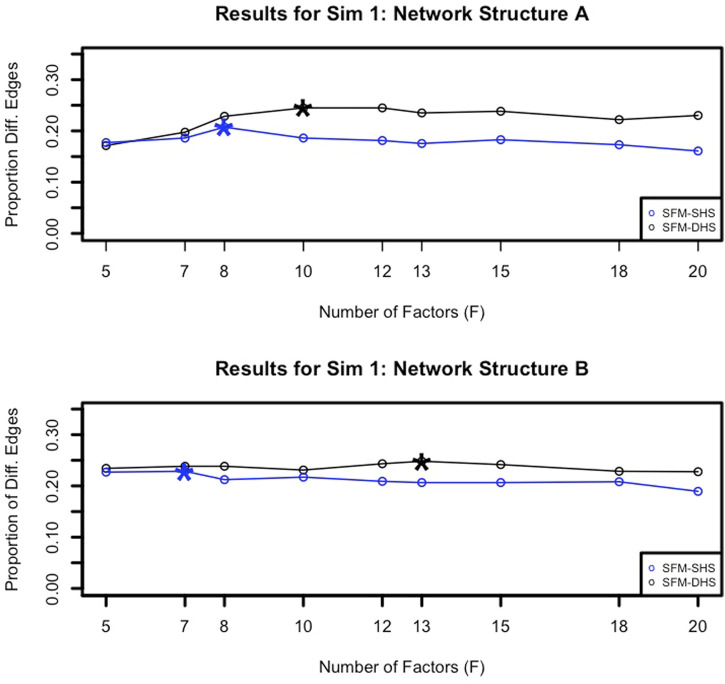
Example proportions of significant differential network edges (number of significant differential network edges divided by $\frac{G\left(G-1\right)}{2}$ ) detected by SFM-SHS and SFM-DHS with bootstrap estimation for different choices of *F* from Sim 1. The ⋆ symbols denote the considered “peak” points across the different number of factor choices.

To identify an appropriate choice for the tuning parameter *F*, the number of factors in our model, we examined the number of significant differential network edges. In Figure 3, we plotted the proportion of differential network edges (number of differential network edges divided by $\frac{G\left(G-1\right)}{2}$ ) determined by both SFM-SHS and SFM-DHS using different choices of *F*. From this figure, we see that the proportion of differential edges tends to increase (as *F* increases) up to a peak and then flattens out or decreases for larger values of *F*. Based on these observations, we determined the “best” model choice for each of our models by identifying the “peak” in the proportion of differential network edges plot after *F* = 5 factors. We focus on the results of the “best” model choice of both SFM-SHS and SFM-DHS for the remainder of this manuscript.

For the simulation studies, we ran also analyses with two other differential network methods that detect differences based on correlation structures between groups. One of the competitor methods we considered was Differential Gene Correlation Analysis (DGCA; McKenzie et al., 2016), which is an R package developed for identifying differential correlations between gene pairs in various types of genomic data (microarray, bulk RNA-seq, scRNA-seq, etc.). DGCA transforms correlation coefficients to z-scores and uses differences in z-scores to generate empirical *p*-values for the differential correlation between genes. These empirical *p*-values are then used to calculate q-values for an FDR threshold, and a q-value of 0.05 was set to denote significant differences for this method.

The other method considered in our simulated studies was scRNA-seq-based differential network analysis (scdNet), which is a differential network method developed specifically for scRNA-seq data by Chiu et al. (2018). In scdNet, a sample size adjustment transformation is first applied to the correlation coefficients within each cellular group and then statistical inference is performed on the differences in the transformed correlations across groups. The scdNet method provides *p*-values to represent differential results for each gene-gene pair, and we controlled the FDR with the Benjamini and Hochberg (1995) procedure. A threshold of 5% was used to indicate significant differences with scdNet.

For each simulated dataset, we compared the significant differences between networks that were identified by each method (SFM-SHS, SFM-DHS, DGCA, and scdNet) to the “true” differences between networks. The measures of TPR, FDR, AUROC, and the number of edges that were classified as significantly different between networks by each method are displayed in Table 1. From this table, we see that our differential network methodology performs quite well compared to the competitor methods of DGCA and scdNet. Both SFM-SHS and SFM-DHS have high TPRs and AUROCs while also controlling the FDRs to a nominal rate. In general, SFM-SHS tends to be a bit more conservative and detects fewer significant edges than SFM-DHS, but SFM-SHS performs better at controlling FDRs below a threshold of 5%. In fact, SFM-SHS was the only method out of the four to consistently control the FDR to a nominal rate across the simulation cases, while scdNet tends to have the highest FDR. The AUROCs for both SFM-SHS and SFM-DHS are also comparable or better than the AUROCs by DGCA and scdNet across most simulations.

To demonstrate the utility of bootstrap estimation compared to the other parameter estimation techniques discussed in Section 2.3, we performed a secondary analysis comparing the results of our methods from bootstrapping to the results of our methods generated by a single optimization and by a full HMC sampler. For the single optimization technique (i.e., finding the posterior mode), the significance of each difference cannot be directly determined; therefore, we identified the Top 10% of edges with the largest difference between treatment groups and calculated the AUROC with the magnitudes (absolute values) of the estimated correlation differences. For the full HMC, we utilized rstan and combined results from 4 separate chains, with each chain running 1,000 warmup iterations and 1,000 sampling iterations for a total of 4,000 samples. Due to the slow speed of HMC sampling, only the results from the smallest simulated datasets (Sim 2: *G* = 50 genes and *N* = 500 cells) are presented.

The results in Table 3 highlight the benefits of using optimization and bootstrapping. Both the full HMC sampler and the bootstrap technique obtain high TPRs and AUROCs, but the HMC sampler tends to have higher FDRs and also has very long computational times. The single optimization technique is also able to achieve high AUROC simply by ranking the magnitudes of the correlation differences between groups. Thus, a single optimization could be useful as a method to quickly identify highly differential edges between networks. We note that bootstrap optimizations can be run simultaneously in parallel and by increasing the number of available cores, the computational efficiency of the bootstrap technique will be increased.

**TABLE 3:** Results for SFM-SHS and SFM-DHS when utilizing bootstrap (Boot), HMC, and the Top 10% of differential edges from a single optimization. Times for Boot represent the average time of one posterior optimization of a single resampled dataset; similarly, HMC time is the average time for a single MCMC chain.

Sim 2: *G* = 50, *N* = 500					
**Network Structure A**	**TPR**	**FDR**	**AUROC**	**Diff. Edges**	**Time**
SFM-SHS; Boot, *F* = 7	0.732	0.040	0.942	248	5.6 s
SFM-SHS; HMC, *F* = 7	0.908	0.366	0.912	465	3.2 days
SFM-SHS; Top 10%, *F* = 7	0.354	0.065	0.803	123^a^	4.9 s
SFM-DHS; Boot, *F* = 8	0.840	0.099	0.946	303	75.6 s
SFM-DHS; HMC, *F* = 8	0.840	0.000	0.984	273	4.6 days
SFM-DHS; Top 10%, *F* = 8	0.369	0.024	0.910	123^a^	18.0 s
**Network Structure B**					
SFM-SHS; Boot, *F* = 7	0.874	0.004	0.979	285	8.0 min
SFM-SHS; HMC, *F* = 7	0.975	0.076	0.985	343	2.7 days
SFM-SHS; Top 10%, *F* = 7	0.366	0.033	0.928	123^a^	16.8 min
SFM-DHS; Boot, *F* = 12	0.855	0.045	0.963	291	11.0 min
SFM-DHS; HMC, *F* = 12	0.849	0.148	0.922	324	5.1 days
SFM-DHS; Top 10%, *F* = 12	0.375	0.008	0.933	123^a^	17.8 min

a Number of edges are fixed.

### 3.2 Case Study

To further examine our proposed methodology, we analyzed the real dataset from Bacher et al. (2020), which consists of scRNA-seq expressions derived from SARS-CoV-2-reactive memory T cells. This data was obtained from the Gene Expression Omnibus (GEO) database under accession number GSE162086. We examined 1,833 cells that were identified by Bacher et al. (2020) as cells with distinct transcriptional profiles related to cytotoxic-Th1 and cycling. These cells are divided into two patient groups: *N*_0_ = 462 cells are from non-hospitalized patients with SARS-CoV-2 and the remaining *N*_1_ = 1, 371 cells come from patients hospitalized with SARS-CoV-2. After filtering out genes that were not expressed in at least 20% of the cells, we used the R package MAST (Finak et al., 2015) to identify *G* = 130 differentially expressed genes with a log2 fold change (as estimated by MAST) of at least log _2_(1.2) for further analysis.

**FIGURE 4:**
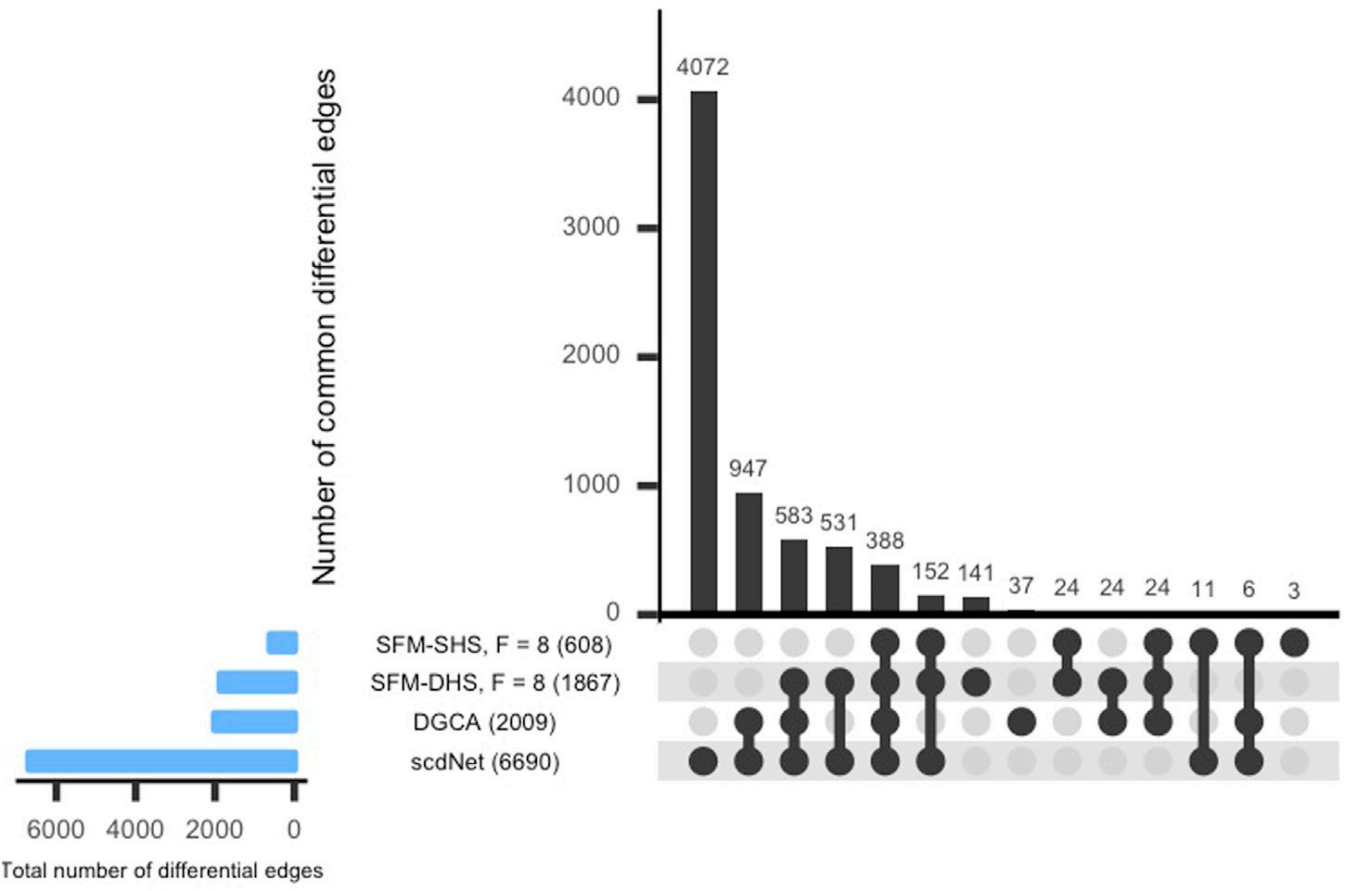
UpSet plot of the number of differential edges determined by four differential network methods in the SARS-CoV-2 case study dataset. The numbers in parentheses represent the total number of differential edges detected by the corresponding method. Results displayed for SFM-SHS and SFM-DHS are from the bootstrap estimation procedure.

For each method considered in Section 3.1, we conducted a differential network analysis and ranked each gene by the number of significant differential connections. SFM-SHS and SFM-DHS were implemented with the bootstrapping estimation approach, and we selected the “best” model choice by identifying the “peak” in the number of differential edges starting with *F* = 5 factors and increasing the number of factors up to *F* = 20. The UpSet plot (Lex et al., 2014) for the intersection between the differential edges detected by each method is displayed in Figure 4. The dark circles in each column of the UpSet plot indicate the methods associated with the intersection and the bar above each column represents the number of differential edges in the intersection.

From Figure 4, we see that the methods performed quite differently as only 388 edges were common across all four methods. SFM-SHS was the most conservative method and detected 608 differential edges, while scdNet method detected 6,690 differential edges, which is nearly 80% of the 8,385 total number of possible edges. Because the differential network analyses from the methods were so different, we selected the top genes with the most gene-gene pair connections from each method and used those top differentially connected genes (DCGs) to evaluate the performance of the methods in this case study analysis. A total of 14 DCGs (approximately 10% of the *G* = 130 total genes) were identified from each of the SFM-SHS, SFM-DHS, and DGCA analyses. From the scdNet analysis, 15 DCGs were chosen because three genes tied for the 13th, 14th, and 15th ranks.

**FIGURE 5:**
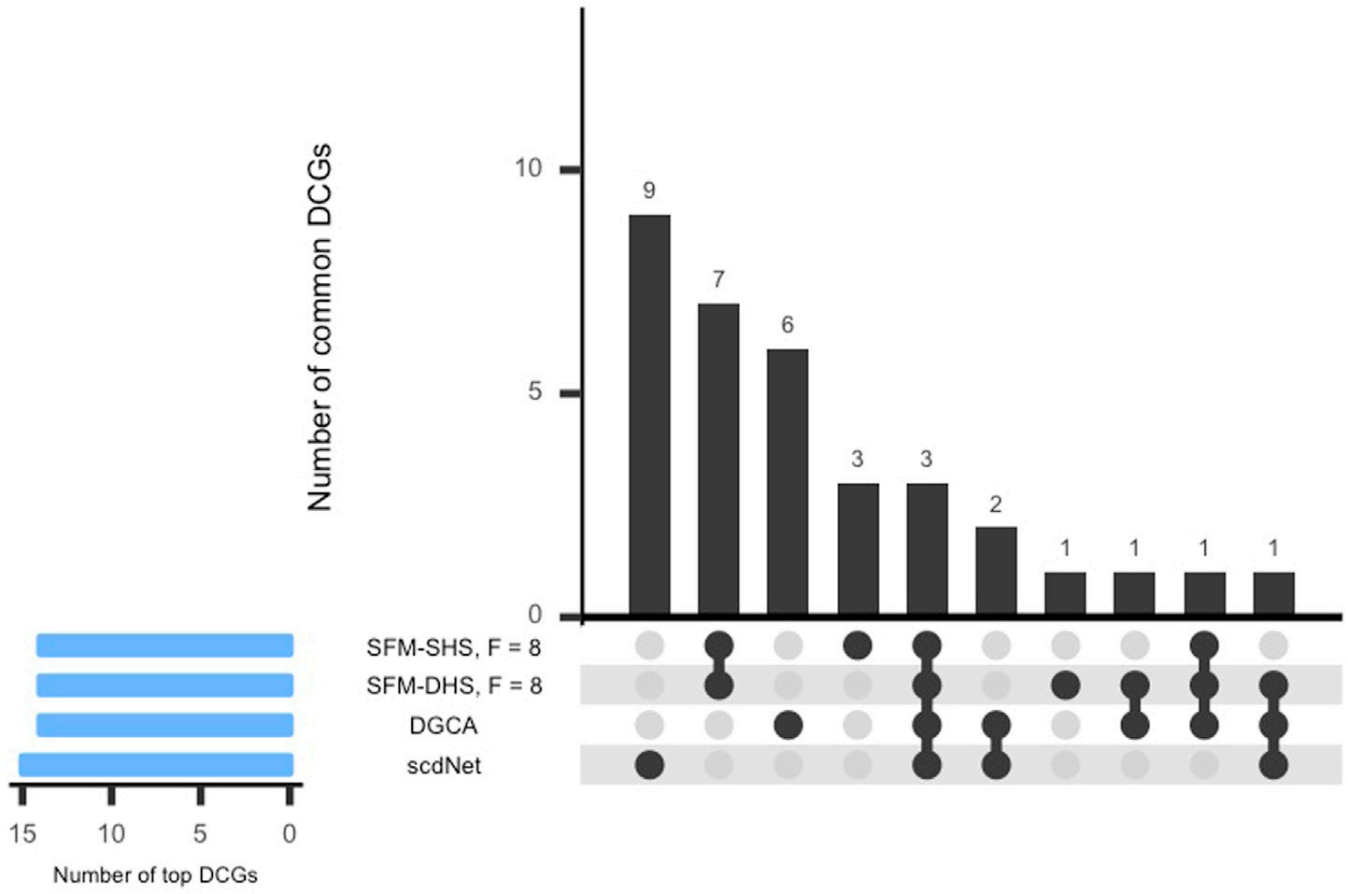
UpSet plot of the top DCGs determined by four differential network methods in the SARS-CoV-2 case study dataset. Results displayed for SFM-SHS and SFM-DHS are from the bootstrap estimation procedure.

In Figure 5, the UpSet plot for the intersection between the top DCGs detected by each differential network method is displayed. Here, the bar above each column in the figure represents the number of DCGs in the intersection. Interestingly, there was not much overlap in the top DCGs detected by the methods considered in this analysis. Our methods (SFM-SHS, SFM-DHS) detect seven unique DCGs from the other methods, whereas DGCA and scdNet identified six and nine unique DCGs, respectively.

Only three genes (*RPS26*, *RGCC*, *RPL3*) were common among the top DCGs selected by each method. These three genes play an important role in any immune-related disorders. *RPS26* and *RPL3* are ribosomal proteins (RP) and both are related to influenza viral RNA transcription and replication. RPs are needed for protein biosynthesis of viruses controlling replication, regulation, and infection inside the host cells. However, a small percentage of these proteins trigger the immune pathway against viruses and protect the host cells. Hence, RPs are now being considered for potential therapeutics for SARS-CoV-2 or any such viral infections (Rofeal and El-Malek, 2020).

To evaluate the biological relevancy of the top DCGs from each method, clusters of gene ontology (GO) categories were created with the functional annotation clustering tool from the database for Annotation, Visualization, and Integrated Discovery (DAVID; Huang et al., 2009a; Huang et al., 2009b). An enrichment score is calculated by DAVID for each cluster to help identify clusters that are involved in more enriched (important) biological roles. As it has been suggested that more attention should be given to groups with enrichment scores greater of 1.3 or higher (Huang et al., 2009b), we used a score threshold of 1.3 to classify clusters as enriched.

SFM-SHS had two enriched clusters from the DAVID functional annotation clustering analysis, while the three other methods (SFM-DHS, DGCA, and scdNet) had just one enriched cluster. SFM-DHS had the cluster with the highest Enrichment Score (3.46), and the top GO terms associated this cluster include SRP-dependent cotranslational protein targeting to membrane, viral transcription, and nuclear-transcribed mRNA catabolic process, nonsense-mediated decay. These same GO terms also appear in the most highly enriched cluster from each of the other three methods, and the corresponding Enrichment Scores from the SFM-SHS, scdNet, and DGCA clusters are 2.95, 2.94, and 2.80, respectively. The DCGs identified by SFM-SHS were also associated with a second enriched cluster. The terms of cytosol, cytoplasm, and phosphoprotein were clustered together with an Enrichment Score of 1.71. All results from DAVID functional annotation clustering tool are provided in the Supplementary Materials.

**FIGURE 6:**
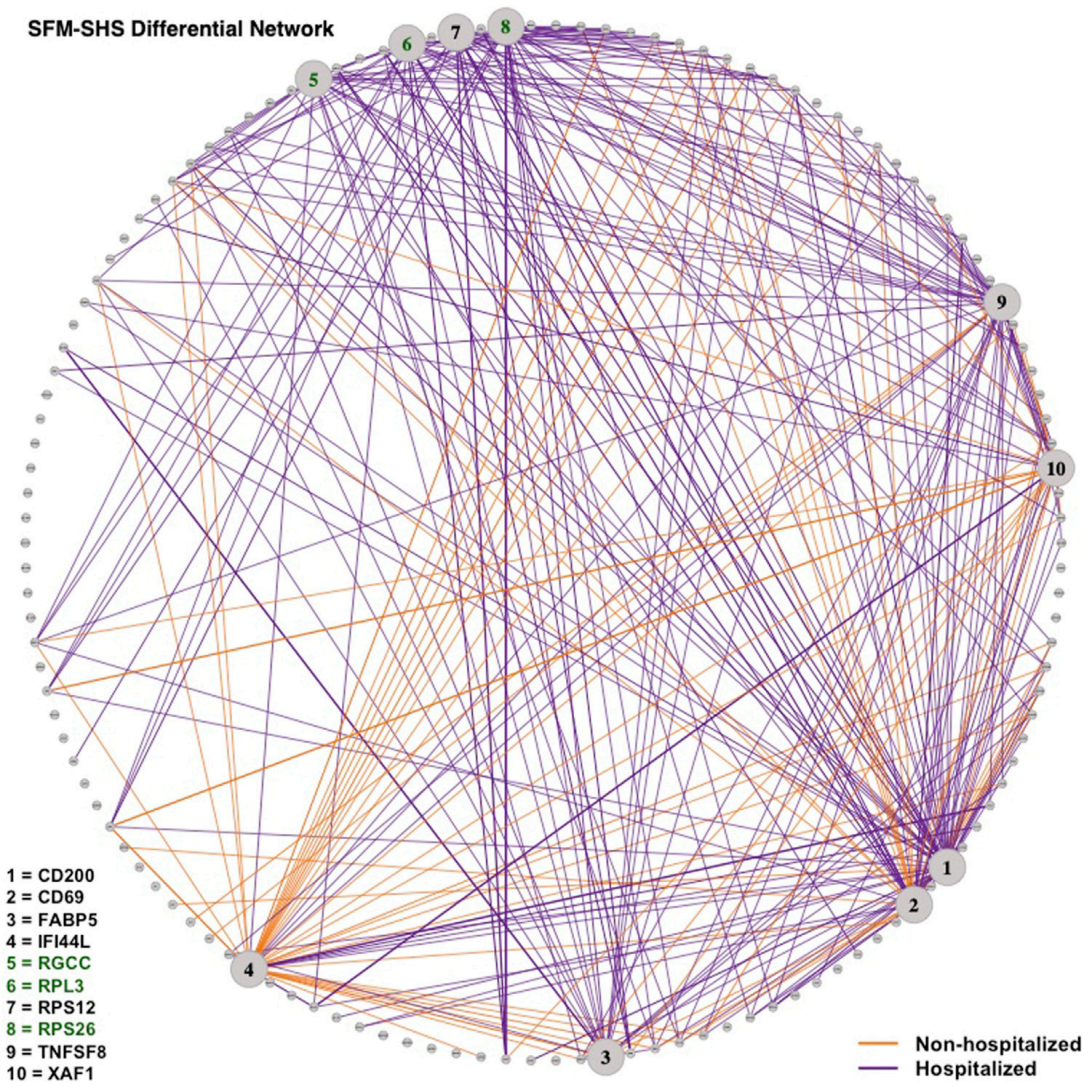
Differential network estimated by SFM-SHS with the bootstrap procedure for the SARS-CoV-2 case study dataset. Edges displayed are for 10 DCGs. The 7 unique top DCGs identified by both SFM-SHS and SFM-DHS are listed in black and the 3 top DCGs identified by all considered methods are listed in green. Each edge color represents the group with the larger correlation magnitude.

**FIGURE 7:**
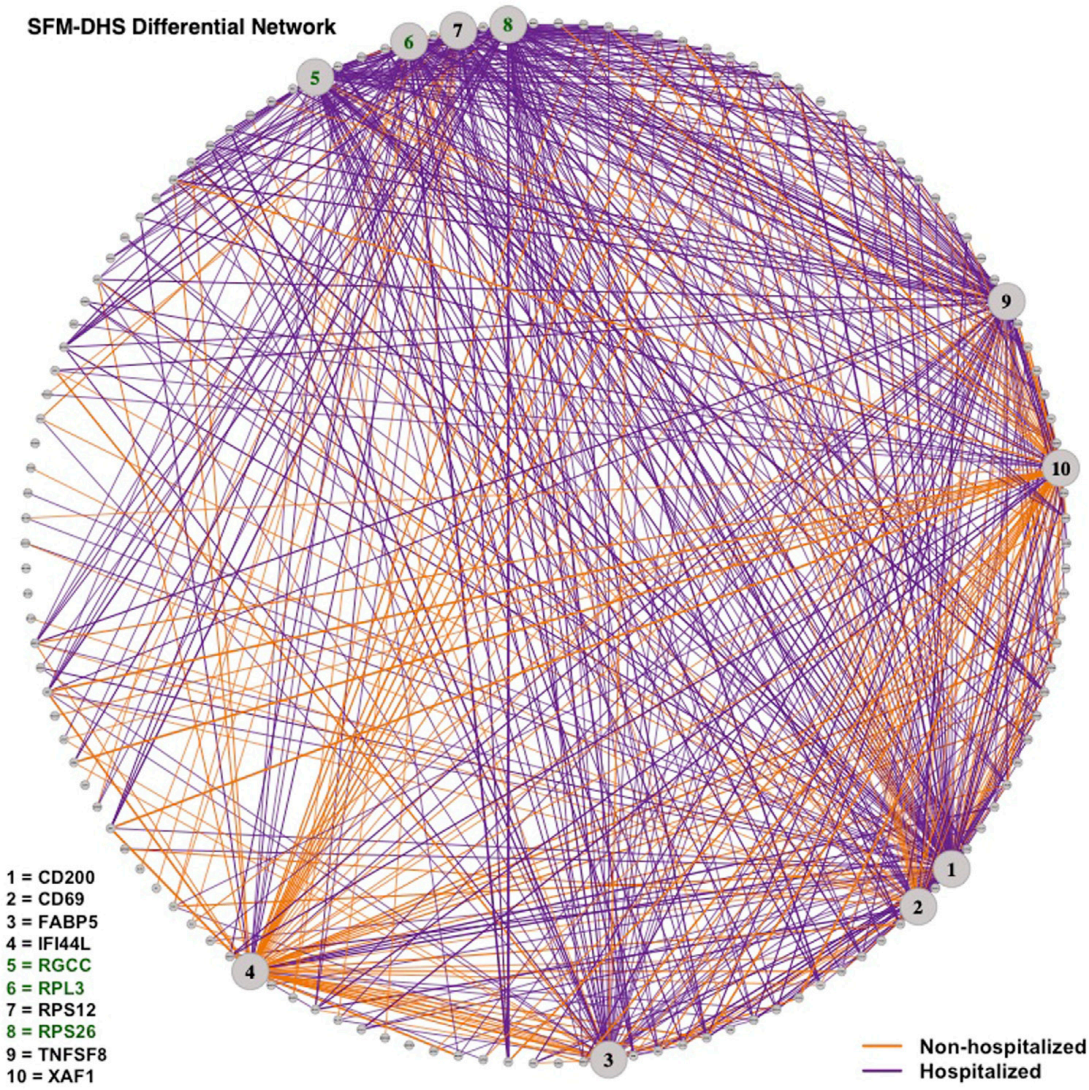
Differential network estimated by SFM-DHS with the bootstrap procedure for the SARS-CoV-2 case study dataset. Edges displayed are for 10 DCGs. The 7 unique top DCGs identified by both SFM-SHS and SFM-DHS are listed in black and the 3 top DCGs identified by all considered methods are listed in green. Each edge color represents the group with the larger correlation magnitude.

Lastly, we visualized the differential networks estimated from SFM-SHS and SFM-DHS in Figures 6, 7, respectively. The gene-gene connections in the figures represent the differential edges for the 7 top DCGs uniquely identified by our methods and the 3 top DCGs identified by all four considered methods. All *G* = 130 genes are displayed in each figure, but we only display the differential edges for the 10 selected DCGs. Figures of the individual GCNs with these 10 DCGs for the non-hospitalized and hospitalized groups are provided in the Supplementary Figures S1–S4.

We see that a majority of these DCGs have differential edges that are a result of larger correlations in the hospitalized group, with *CD200* and *TNFSF8* having the highest numbers of differential edges from this group. *CD200* is a type 1 cell membrane glycoprotein (GP) of the immunoglobulin supergene family that is expressed by many cell types (e.g., B cells, a subset of T cells, endothelial cells, cancer cells). GP plays an important role in immunosuppression and regulation of anti-tumor activity. Moreover, this gene has multiple transcript variances. Naturally, its connectivities are different in the hospitalized group of patients compared to non-hospitalized group.

*TNFSF8* is one of the tumor necrosis factor (TNF) receptor superfamily proteins that typically are composed of one to four cysteine-rich domains. It has been suggested that SARS-CoV-2 disease processes present in severe illness contribute to impaired adaptive immune responses. Additionally, TNF super family proteins are most often used in predicting neutralization. Elderly patients severely affected by SARS-CoV-2 have distinctive neutralization activity-associated protein profiles that may display an altered level of *TNFSF8* (Filbin et al., 2021).

Conversely, both *IFI44L* and *XAF1* had the highest number of differential edges as a result of having stronger correlations in the non-hospitalized group. Prior studies concluded that SARS-CoV-2 and other viruses elicit an interferon response in the upper airway. Moreover, the most significant genes upregulated by SARS-CoV-2 were interferon inducible, such as *IFI44L* (Butler et al., 2021; Huang et al., 2021). *XAF1* is an X-linked inhibitor of apoptosis (XIAP)-associated factor 1. This gene participates in pro-apoptotic responses and has multiple transcript variants. In Zhu et al. (2020), both *IFI44L* and *XAF1* were upregulated in T, B, natural killer, and DC cell subsets of SARS-CoV-2 patients compared to healthy controls.

## 4 Discussion

Our proposed model includes continuous treatment-dependent parameters that determine the impact of latent factors for each gene. For simplicity, our methodology has been defined and examined under a two-group situation, but it can be adjusted to a multiple group scenario. In the general case, we can consider *T* number of treatments and represent the *log*(*μ*_*gi*_) from Eq. 1 in the general form:

$$\log\left(\mu_{gi}\right)=\beta_{g}+\overset{T-1}{\underset{t=1}{\sum }}I\left(t_{i}=t\right)\delta_{g;t}+\overset{F}{\underset{f=1}{\sum }}\lambda_{if}\alpha_{gf;t_{i}}-\left\{\overset{F}{\underset{f=1}{\sum }}\frac{\alpha_{gf;t_{i}}^{2}}{2}\right\}.$$

Here, the *δ*_*g*;*t*_ parameters depend on the treatment groups *t* ∈ {1, *…*, *T* − 1} and *I*(*t*_*i*_ = *t*) is the indicator variable for cell *i* being in treatment group *t*. The construction of gene-gene correlation structures will remain the same, but there will be *T* sets of *α*_*gf*;*t*_ parameters that create *T* different networks to compare. When performing differential network analysis, one can examine the CrIs (or CIs) of the difference between *ρ*_*gg*′;*t*_ and *ρ*_*gg*′;*t*′_ for each pair of treatments *t* and *t*′ (*t* ≠ *t*′).

In addition to identifying differential network edges, our methodology also reports estimates of the GCNs for both the control and treatment group. We found that these estimates generally reflect the “true” underlying correlation structures of the simulated datasets used in Section 3.1 (see Supplementary Tables S1, S2). To the best of our knowledge, the method of scdNet currently does not directly provide the estimations of the control and treatment GCNs, separately.

When applying our methodology, we recommend using an optimization-based procedure for estimating model parameters. Researchers can choose to utilize a single optimization of our model and select the “Top *N*” gene-gene correlation differences from the posterior mode or choose to utilize bootstrapping to obtain and analyze the variability of the estimates. Both techniques achieved high AUROCs in the simulation studies, and the bootstrap optimization was able to control the FDRs. Furthermore, bootstrap replicates can be performed in parallel to help reduce computational time. One could choose to utilize a full HMC to obtain parameter estimates and perform model inference, but an iterative MCMC approach may be computationally expensive and will also require the use of diagnostic tools to assess convergence. As an alternative approach to faster Bayesian computing, we had considered utilizing variational inference as a tool to approximate the posterior distribution and obtain parameter estimates as in Sekula et al. (2019), but our preliminary experiments found that this inference technique did not produce reliable estimates.

When it comes to choosing an appropriate number of factors (*F*) for our methodology, we recommend running different choices for *F* and identifying the “peak” in the number of differential network edges after *F* = 5 factors (as illustrated in Figure 3). Starting with the choice of *F* = 5 helps to capture the increase in the number of differential edges across increasing values of *F*, and the number of factors associated with the “peak” can be considered the “best” model choice. Generally, we found that *F* = 7 or *F* = 8 for the number of factors was a common selection for both SFM-SHS and SFM-DHS in our considered datasets that had between *G* = 50 to *G* = 130 genes. Thus, using either *F* = 7 or *F* = 8 would be a reasonable default choice for analyses with similar numbers of genes. For analyses with much larger values of *G*, one may anticipate needing a larger value for *F*.

## 5 Conclusion and Future Scope

In this manuscript, we have presented a sparse hierarchical Bayesian factor model to perform differential network analysis from scRNA-seq count data. With a latent factor structure, we define a count model that is conditionally Poisson but marginally overdispersed, and the flexibility of the defined latent factor structure allows our model to account for other unique features of scRNA-seq data such as zero-inflation and high cell-to-cell variability. Furthermore, the defined horseshoe prior structures in Eqs. 2–4 promote sparsity in our network estimation and allow information to be shared across treatment groups.

When applying our methodology, our main recommendation is to perform analysis using the SHS version of our model with bootstrapping, as SFM-SHS tends to be better at controlling the FDRs compared to SFM-DHS. We also recommend using some sort of gene selection method (e.g., differential expression) to obtain a manageable number of genes to analyze with our method. Since each GCN is determined by a quadratic number of parameters ( $\frac{G\left(G-1\right)}{2}$ correlations), it becomes difficult to present results both visually and numerically for larger values of *G*.

As demonstrated in Section 3.1, the bootstrapping technique for parameter estimation provides a time-efficient implementation of our methodology that outperforms the competitor methods of scdNet and DGCA. Also, our methods were superior in selecting top DCGs that were associated with biologically enriched clusters of GO categories in Section 3.2. Both SFM-SHS and SFM-DHS identified a unique set of top DCGs with biological functions related to the body’s response to the SARS-CoV-2 virus. Collectively, these analyses suggest that our sparse Bayesian factor model will be a useful tool for the construction and differential analysis of GCNs in future scRNA-seq experiments. An R package implementing our proposed methodology and code to generate the simulated datasets are available at: https://github.com/mnsekula/scSFMnet.

## Data Availability

Publicly available datasets were analyzed in this study. This data can be found here: https://www.ncbi.nlm.nih.gov/geo/query/acc.cgi?acc=GSE162086.
