# Supplementary material for: Single-Cell Differential Network Analysis with Sparse Bayesian Factor Models
Source: Front Genet. 2022 Feb 4;12:810816. doi: 10.3389/fgene.2021.810816 (PMC8855158; doi:10.3389/fgene.2021.810816)
Supplement: Supplementary file 1 [file DataSheet1.pdf]

Supplementary Material

Single-cell Differential Network Analysis with Sparse Bayesian Factor Models

1 SUPPLEMETARY FIGURES

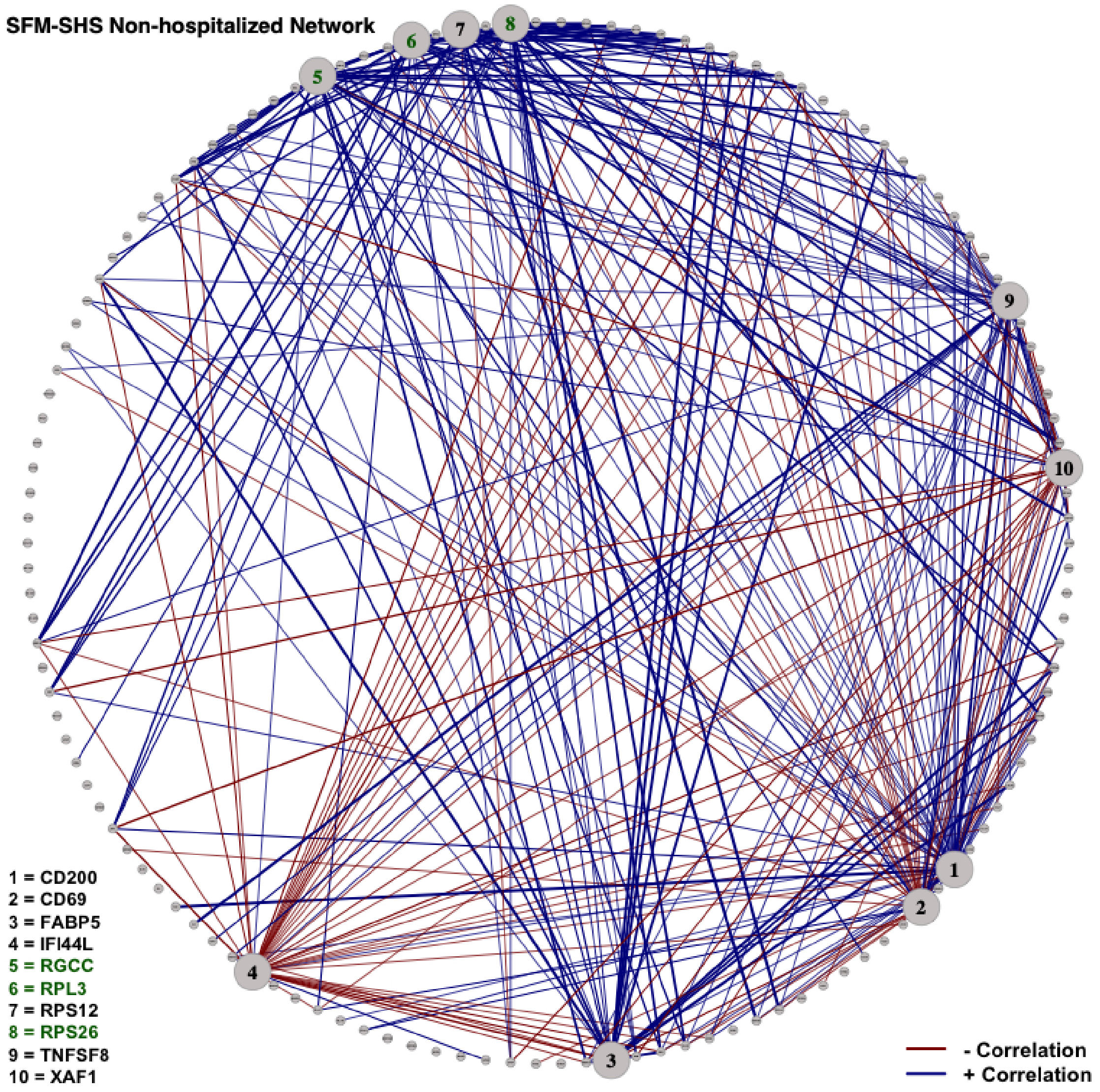

Figure S1. Network estimated by SFM-SHS with the bootstrap procedure for the non-hospitalized group in the SARS-CoV-2 case study dataset. Edges displayed are for 10 DCGs. The 7 unique top DCGs identified by both SFM-SHS and SFM-DHS are listed in black and the 3 top DCGs identified by all considered methods are listed in green. Each edge is colored to represent the direction of correlation.

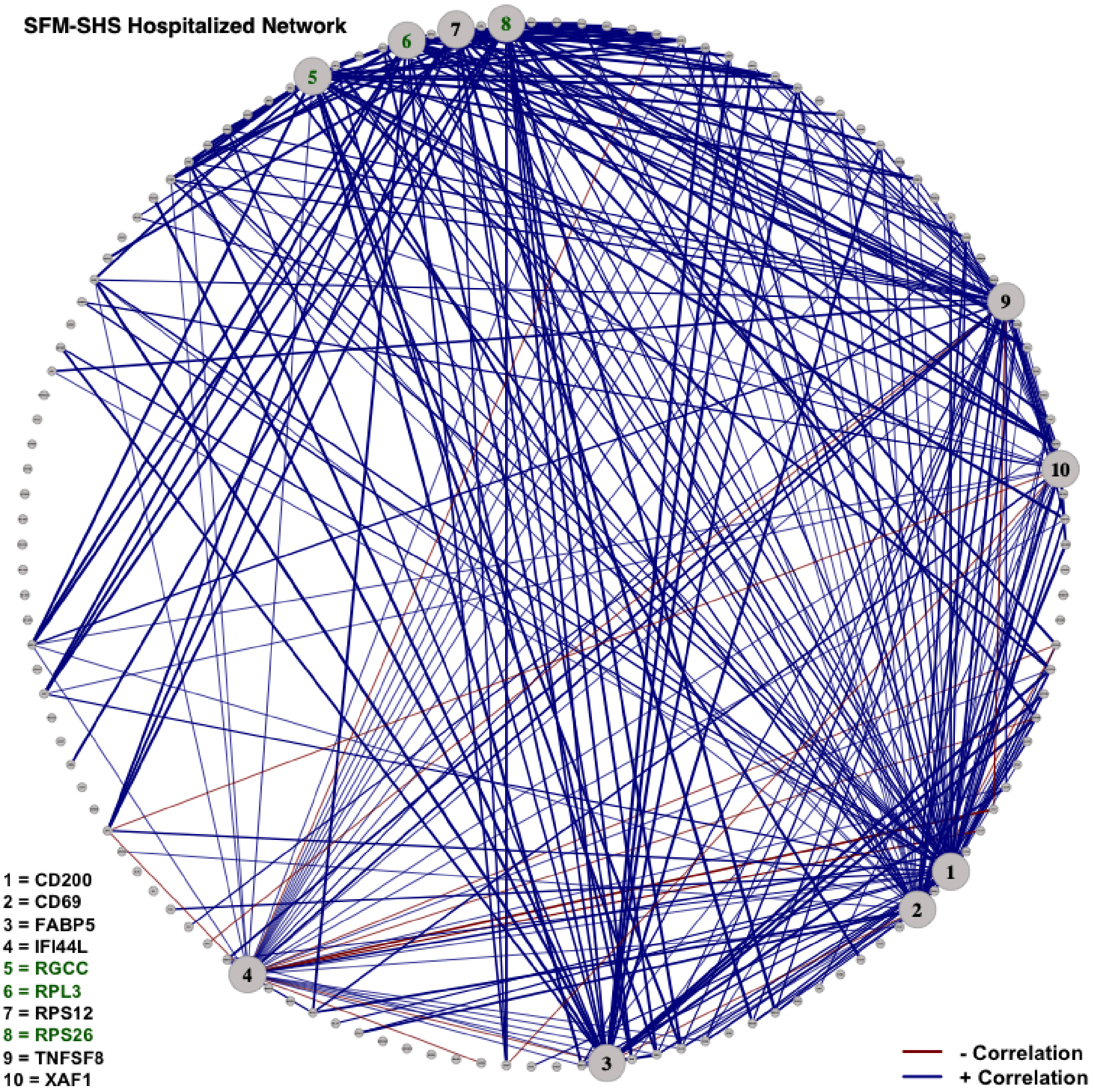

Figure S2. Network estimated by SFM-SHS with the bootstrap procedure for the hospitalized group in the SARS-CoV-2 case study dataset. Edges displayed are for 10 DCGs. The 7 unique top DCGs identified by both SFM-SHS and SFM-DHS are listed in black and the 3 top DCGs identified by all considered methods are listed in green. Each edge is colored to represent the direction of correlation.

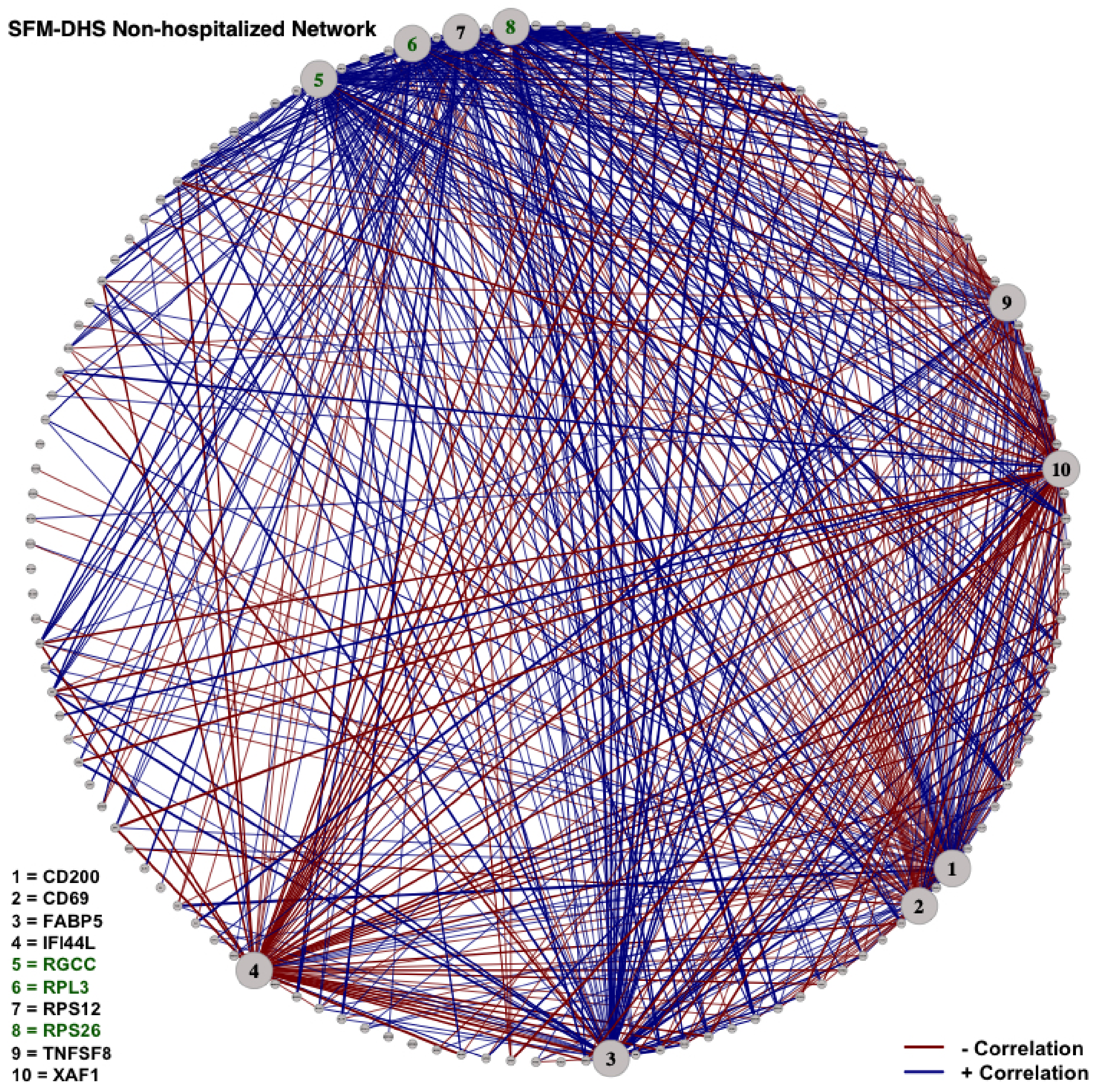

Figure S3. Network estimated by SFM-DHS with the bootstrap procedure for the non-hospitalized group in the SARS-CoV-2 case study dataset. Edges displayed are for 10 DCGs. The 7 unique top DCGs identified by both SFM-SHS and SFM-DHS are listed in black and the 3 top DCGs identified by all considered methods are listed in green. Each edge is colored to represent the direction of correlation.

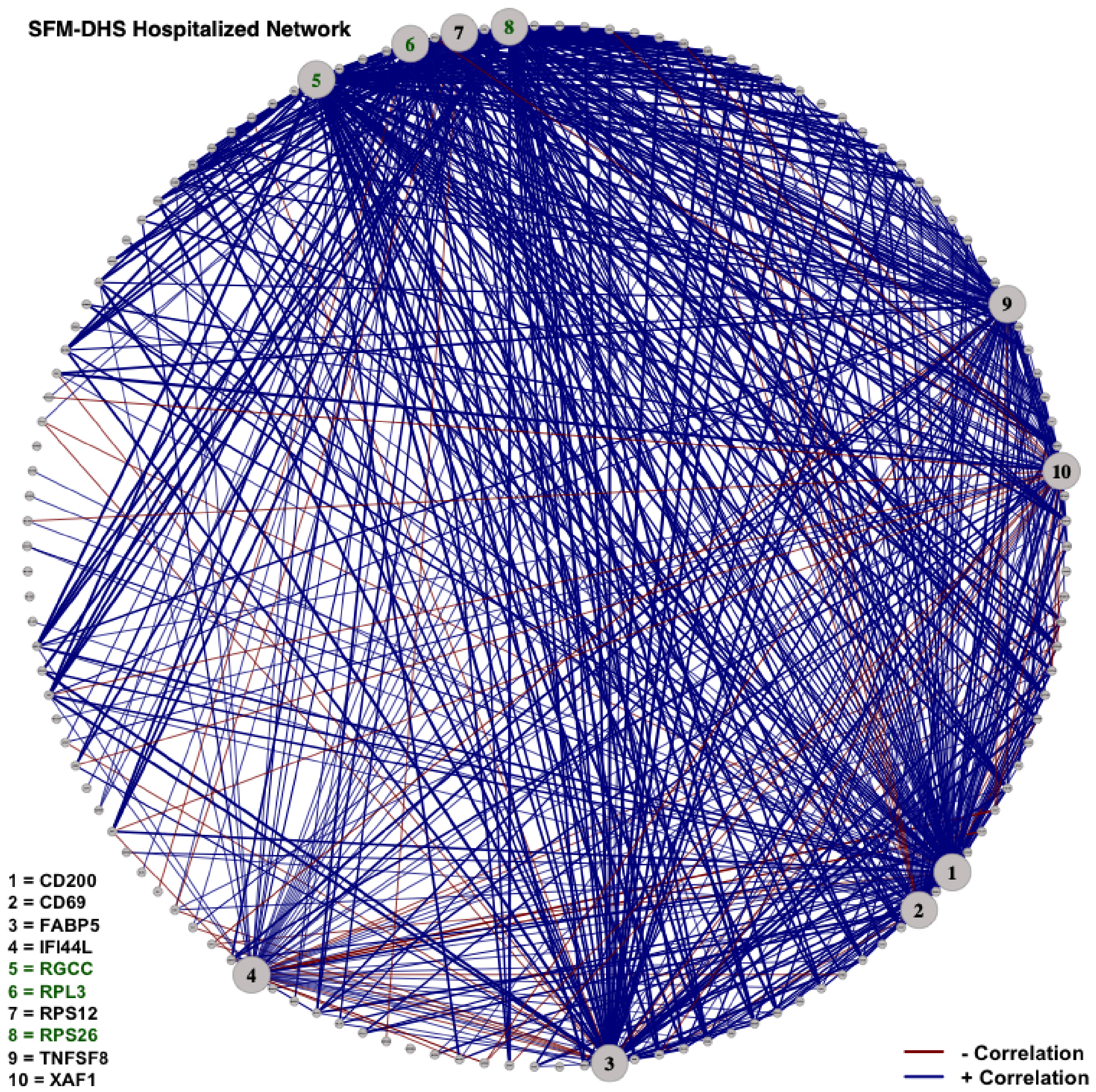

Figure S4. Network estimated by SFM-DHS with the bootstrap procedure for the hospitalized group in the SARS-CoV-2 case study dataset. Edges displayed are for 10 DCGs. The 7 unique top DCGs identified by both SFM-SHS and SFM-DHS are listed in black and the 3 top DCGs identified by all considered methods are listed in green. Each edge is colored to represent the direction of correlation.

2 SUPPLEMENTARY TABLES

**Sim 1:** $G = 50, N = 1000$				
**Network Structure A**	TPR_0	FDR_0	$AUROC_0$	$Edges_0$
**SFM-SHS;** $F = 8$	0.766	0.082	0.978	292
**SFM-DHS;** $F = 10$	0.817	0.154	0.960	338
**DGCA**	0.906	0.086	0.983	347
**Network Structure B**				
**SFM-SHS;** $F = 7$	0.914	0.056	0.983	339
**SFM-DHS;** $F = 13$	0.923	0.225	0.942	417
**DGCA**	0.937	0.000	0.985	328
**Sim 2:** $G = 50, N = 500$				
**Network Structure A**	TPR_0	FDR_0	$AUROC_0$	$Edges_0$
**SFM-SHS;** $F = 7$	0.814	0.072	0.987	307
**SFM-DHS;** $F = 8$	0.840	0.133	0.980	339
**DGCA**	0.911	0.000	0.997	319
**Network Structure B**				
**SFM-SHS;** $F = 7$	0.951	0.015	0.995	338
**SFM-DHS;** $F = 12$	0.931	0.133	0.978	376
**DGCA**	0.960	0.034	0.991	348
**Sim 3:** $G = 50, N = 2,000$				
**Network Structure A**	TPR_0	FDR_0	$AUROC_0$	$Edges_0$
**SFM-SHS;** $F = 8$	0.863	0.000	0.995	302
**SFM-DHS;** $F = 8$	0.914	0.083	0.983	349
**DGCA**	0.931	0.175	0.979	395
**Network Structure B**				
**SFM-SHS;** $F = 7$	0.951	0.067	0.998	357
**SFM-DHS;** $F = 8$	0.991	0.202	0.987	435
**DGCA**	0.977	0.128	0.997	392
**Sim 4:** $G = 100, N = 1,000$				
**Network Structure A**	TPR_0	FDR_0	$AUROC_0$	$Edges_0$
**SFM-SHS;** $F = 8$	0.918	0.000	0.998	1331
**SFM-DHS;** $F = 8$	0.921	0.123	0.975	1523
**DGCA**	0.921	0.016	0.985	1358
**Network Structure B**				
**SFM-SHS;** $F = 7$	0.987	0.120	0.991	1627
**SFM-DHS;** $F = 10$	0.974	0.259	0.954	1906
**DGCA**	0.981	0.003	0.998	1426

Table S1. Comparison of the "true" control network structure and the estimated network structure in the simulation studies for SFM-SHS, SFM-DHS, and DGCA. Results displayed for SFM-SHS and SFM-DHS are from the bootstrap estimation procedure. The method of scdNet does not provide directly provide this estimation. In Sim 1 - 3, there are 350 "true" control network edges and in Sim 4 there are 1,450 "true" control network edges.

**Sim 1: $G = 50, N = 1000$**				
**Network Structure A**	TPR_1	FDR_1	$AUROC_1$	$Edges_1$
**SFM-SHS; $F = 8$**	0.812	0.090	0.979	290
**SFM-DHS; $F = 10$**	0.883	0.201	0.957	359
**DGCA**	0.902	0.039	0.953	305
**Network Structure B**				
**SFM-SHS; $F = 7$**	0.969	0.003	1.000	316
**SFM-DHS; $F = 13$**	0.957	0.063	0.993	332
**DGCA**	0.957	0.091	0.992	342
**Sim 2: $G = 50, N = 500$**				
**Network Structure A**	TPR_1	FDR_1	$AUROC_1$	$Edges_1$
**SFM-SHS; $F = 7$**	0.822	0.000	0.994	267
**SFM-DHS; $F = 8$**	0.840	0.199	0.954	341
**DGCA**	0.905	0.010	0.941	297
**Network Structure B**				
**SFM-SHS; $F = 7$**	0.911	0.003	0.998	297
**SFM-DHS; $F = 12$**	0.908	0.007	0.993	297
**DGCA**	0.966	0.003	0.989	315
**Sim 3: $G = 50, N = 2,000$**				
**Network Structure A**	TPR_1	FDR_1	$AUROC_1$	$Edges_1$
**SFM-SHS; $F = 8$**	0.822	0.133	0.984	308
**SFM-DHS; $F = 8$**	1.000	0.201	0.970	407
**DGCA**	0.991	0.108	0.998	361
**Network Structure B**				
**SFM-SHS; $F = 7$**	0.902	0.064	0.985	313
**SFM-DHS; $F = 8$**	1.000	0.074	0.988	351
**DGCA**	0.994	0.058	0.998	343
**Sim 4: $G = 100, N = 1,000$**				
**Network Structure A**	TPR_1	FDR_1	$AUROC_1$	$Edges_1$
**SFM-SHS; $F = 8$**	0.840	0.038	0.988	1179
**SFM-DHS; $F = 8$**	0.841	0.251	0.926	1517
**DGCA**	0.973	0.077	0.988	1423
**Network Structure B**				
**SFM-SHS; $F = 7$**	0.961	0.009	0.999	1310
**SFM-DHS; $F = 10$**	0.923	0.116	0.985	1410
**DGCA**	0.982	0.019	0.997	1352

Table S2. Comparison of the "true" treatment network structure and the estimated network structure in the simulation studies for SFM-SHS, SFM-DHS, and DGCA. Results displayed for SFM-SHS and SFM-DHS are from the bootstrap estimation procedure. The method of scdNet does not provide directly provide this estimation. In Sim 1 - 3, there are 325 "true" treatment network edges and in Sim 4 there are 1,350 "true" treatment network edges.
